# A Combined Chronic Low-Dose Soluble Epoxide Hydrolase and Acetylcholinesterase Pharmacological Inhibition Promotes Memory Reinstatement in Alzheimer’s Disease Mice Models

**DOI:** 10.3390/ph15080908

**Published:** 2022-07-22

**Authors:** Júlia Jarne-Ferrer, Christian Griñán-Ferré, Aina Bellver-Sanchis, Santiago Vázquez, Diego Muñoz-Torrero, Mercè Pallàs

**Affiliations:** 1 Department of Pharmacology, Toxicology and Therapeutic Chemistry, Institut de Neurociències-Universitat de Barcelona, Avenida Joan XXIII, 27-31, E-08028 Barcelona, Spain; jfjulia97@gmail.com (J.J.-F.); christian.grinan@ub.edu (C.G.-F.); abellversanchis@gmail.com (A.B.-S.); 2 CSIC Associated Unit, Laboratory of Medicinal Chemistry, Faculty of Pharmacy and Food Sciences, Institute of Biomedicine (IBUB), University of Barcelona, Avenida Joan XXIII, 27-31, E-08028 Barcelona, Spain; svazquez@ub.edu (S.V.); dmunoztorrero@ub.edu (D.M.-T.)

**Keywords:** soluble epoxide hydrolase, acetylcholinesterase, cognitive decline, Alzheimer’s disease, neuroinflammation, multitarget agents, neurodegeneration

## Abstract

Alzheimer’s disease (AD) is a progressive neurological disorder with multifactorial and heterogeneous causes. AD involves several etiopathogenic mechanisms such as aberrant protein accumulation, neurotransmitter deficits, synaptic dysfunction and neuroinflammation, which lead to cognitive decline. Unfortunately, the currently available anti-AD drugs only alleviate the symptoms temporarily and provide a limited therapeutic effect. Thus, new therapeutic strategies, including multitarget approaches, are urgently needed. It has been demonstrated that a co-treatment of acetylcholinesterase (AChE) inhibitor with other neuroprotective agents has beneficial effects on cognition. Here, we have assessed the neuroprotective effects of chronic dual treatment with a soluble epoxide hydrolase (sEH) inhibitor (TPPU) and an AChE inhibitor (6-chlorotacrine or rivastigmine) in in vivo studies. Interestingly, we have found beneficial effects after chronic low-dose co-treatment with TPPU and 6-chlorotacrine in the senescence-accelerated mouse prone 8 (SAMP8) mouse model as well as with TPPU and rivastigmine co-treatment in the 5XFAD mouse model, in comparison with the corresponding monotherapy treatments. In the SAMP8 model, no substantial improvements in synaptic plasticity markers were found, but the co-treatment of TPPU and 6-chlorotacrine led to a significantly reduced gene expression of neuroinflammatory markers, such as interleukin 6 (*Il-6*), triggering receptor expressed on myeloid cell *2* (*Trem2*) and glial fibrillary acidic protein (*Gfap*). In 5XFAD mice, chronic low-dose co-treatment of TPPU and rivastigmine led to enhanced protein levels of synaptic plasticity markers, such as the phospho-cAMP response element-binding protein (p-CREB) ratio, brain-derived neurotrophic factor (BDNF) and postsynaptic density protein 95 (PSD95), and also to a reduction in neuroinflammatory gene expression. Collectively, these results support the neuroprotectant role of chronic low-dose co-treatment strategy with sEH and AChE inhibitors in AD mouse models, opening new avenues for effective AD treatment.

## 1. Introduction

The significant advance in modern medicine has led to an increase in the life expectancy of human beings. Consequently, there has been an increase in the prevalence of many age-related diseases, such as neurodegenerative diseases [1]. Nowadays, more than a hundred neurodegenerative diseases are of utmost importance due to their incidence and/or severity, as is the case of Alzheimer’s disease (AD) [2]. AD is characterized by progressive memory loss and cognitive decline and has become the major neurodegenerative disease in aging populations of modern societies [3]. It is well known that AD’s two key pathogenic hallmarks are the accumulation of extracellular plaques composed of amyloid-β (Aβ) and intracellular neurofibrillary tangles (NFTs), containing hyperphosphorylated Tau proteins [4,5]. However, AD is multifactorial and heterogeneous and involves several etiopathogenic mechanisms [6].

Up to now, there is no effective treatment for AD, and all drugs, namely acetylcholinesterase (AChE) inhibitors and N-methyl-D-aspartate (NMDA) competitive antagonists, are approved for palliative treatment [7]. However, although new therapeutic strategies against Aβ and Tau hyperphosphorylation have been proposed, all have failed so far. Therefore, there is an urgent and vitally important need to search for effective drugs capable of delaying, slowing down and/or reversing neuronal damage and curing neurodegenerative diseases [7].

It is well accepted that neurodegeneration is a consequence of several pathological mechanisms, which results in the loss of neurons and cognition [8]. Thus, an effective treatment should modify neurodegeneration by stopping neuronal death and/or promoting neurons’ functionality, slowing disease progression and relieving clinical symptoms for extended periods. On the one hand, one of the most important neurotransmitter systems affected in age-related cognitive impairment is the cholinergic system. Specifically, an impairment of the cholinergic function is involved in the pathophysiology of AD [9,10]. Hence, ameliorating the cholinergic neurotransmission remains a primary pharmacological strategy for cognitive and behavioral alterations of mild and moderate AD stages [11]. On the other hand, among the novel therapeutic strategies to cope with AD, treatments that target neuroinflammation constitute a very promising approach to rescue neuronal loss and cognitive decline [12]. In this context, the enzyme soluble epoxide hydrolase (sEH) has recently emerged as a very valuable therapeutic target. sEH participates in the degradation of epoxyeicosatrienoic acids (EETs), which are endogenous compounds that derive from the metabolism of arachidonic acid and that are endowed with anti-inflammatory properties [13]. Thus, degradation of EETs by sEH abolishes their beneficial anti-inflammatory effects. Interestingly, it has been reported that sEH is overexpressed in neurological disorders such as depression [14], schizophrenia [15] and Parkinson’s disease (PD) [16], indicating the role of the enzyme in these neurodegenerative processes. Notably, it is widely described that sEH protein levels are increased in the brain of AD patients with Braak II/IV and several AD mouse models [17], including the senescence-accelerated mouse prone 8 (SAMP8) and 5XFAD mice. Besides, genetic ablation of sEH in AD model mice delayed the progression of neurodegeneration [17,18]. Furthermore, sEH inhibitors (sEHi), as TPPU, effectively reduced Aβ plaques, Tau hyperphosphorylation and neuroinflammation markers in both mouse models [12]. Remarkably, the genetic deletion of the *EPHX2* gene or the use of sEHi also showed effectiveness in different in vitro models of neurodegeneration [12]. These results suggest that sEH pharmacological inhibition might be a promising therapy for AD patients.

Very interestingly, the cholinergic system is linked to the production of EETs. Indeed, activating muscarinic M1 receptors by the neurotransmitter acetylcholine (ACh) promotes the metabolism of arachidonic acid to EETs [19,20]. Thus, the increased levels of ACh upon AChE inhibition should lead to increased levels of EETs, and hence, to potentiation of their anti-neuroinflammatory effects. Thus, dual inhibition of sEH and AChE should result in additive or synergistic effects against neuroinflammation, a critical early mechanism of AD, and beneficial effects on cognition. Based on these premises, we have recently demonstrated that a chronic low dose of a dual inhibitor, targeting sEH and AChE (i.e., a multitarget compound), was able to rescue cognitive decline, AD hallmarks and synaptic dysfunction in SAMP8, a mouse model of late-onset AD (LOAD) [19].

Besides multitarget compounds, another more classical modality of multitarget treatment relies on drug combinations. This strategy has proven successful in some complex multifactorial diseases, such as cancer and HIV, among others [21,22]. In the present study, we aimed (i) to explore the potential of a drug combination strategy based on sEH and AChE inhibition; (ii) to further support dual inhibition of sEH and AChE as a new general therapeutic strategy against neuroinflammation and cholinergic deficits; and (iii) to deep into the molecular mechanisms that underlie the beneficial effects promoted by dual treatment. In analogy with our previous work that demonstrated beneficial effects in SAMP8 mice with the aforementioned dual acting TPPU-6-chlorotacrine hybrid compound [19]. Here, we decided to explore a combination based on the same constituting drugs of that hybrid, i.e., TPPU [23] and 6-chlorotacrine (6-Cl-THA) [24], using the same AD mouse model. Besides, to get further insight into the therapeutic potential of the sEHi + AChEi drug combination, we additionally selected a combination of TPPU with a structurally and mechanistically different AChEi, namely the marketed drug rivastigmine (Riv), using a different mouse model of AD. Thus, here, we have explored the effects induced by a chronic low dose treatment of a combination of the sEHi (TPPU), with either the reversible AChEi (6-Cl-THA) or a pseudoirreversible AChEi (Riv), in two different mouse models of AD, SAMP8 and 5XFAD mice. As previously mentioned, SAMP8 is a model of LOAD, which is suitable for studying age-related cognitive impairment associated with a decline of the cholinergic system [25,26] and neuroinflammation [27], whereas 5XFAD is a widely used model of early-onset familial AD (EOFAD) [28].

## 2. Results

### 2.1. TPPU and 6-Cl-THA Chronic Low-Dose Co-Treatment Rescued SAMP8 Cognitive Decline, Ameliorating Neuroinflammation Markers

**Figure 1:**
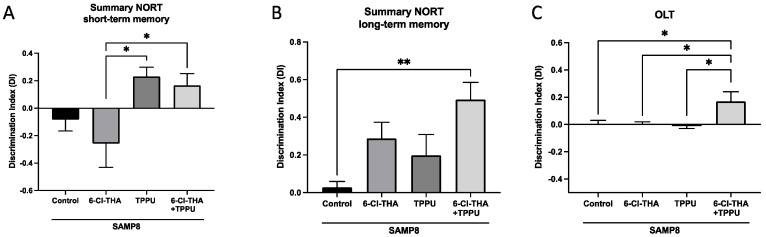
Effects of the drug combination and monotherapies on cognitive performance in SAMP8 mice. (**A**) Short-term memory test at 2 h. (**B**) Long-term memory test at 24 h. (**C**) OLT. Results are expressed as a mean ± SEM. SAMP8 control, *n* = 8; SAMP8 treated with the TPPU (1 mg/kg/day), *n* = 8; SAMP8 treated with 6-Cl-THA (1 mg/kg/day), *n* = 8; SAMP8 subjected to co-treatment with TPPU (1 mg/kg/day) plus 6-Cl-THA (1 mg/kg/day), *n* = 8. Groups were compared by a one-way ANOVA test and post-hoc Tukey’s test. * *p* < 0.05; ** *p* < 0.01. Abbreviations: 6-Cl-THA: 6-chlorotacrine; NORT = novel object recognition test; OLT = object location test.

As mentioned above, SAMP8 presents cognitive decline associated with working and spatial memories. On the one hand, we evaluated working memory through NORT. Regarding the effects on short-term memory, SAMP8 mice treated with the combination performed the test significantly better than mice treated with 6-Cl-THA, and similarly to the group treated with TPPU. Regarding the effects on long-term memory, our results showed a higher DI, and hence, a more potent cognition-enhancing effect in SAMP8 treated with the low dose combination of sEHi (TPPU) + AChEi (6-Cl-THA) than in control mice and in the groups subjected to monotherapy (Figure 1A,B). On the other hand, we also analyzed the spatial memory, measured through OLT. We found that monotherapy treatments with 6-chorotacrine or TPPU did not improve the spatial memory relative to the control group. Gratifyingly, we obtained significant differences in the group treated with the combination sEHi + AChEi compared with SAMP8 control (Figure 1C). Overall, these results suggest a more effective prevention of cognitive decline and a synergistic effect at the cognitive level in the SAMP8 strain by the combination of the sEHi TPPU plus the reversible AChEi 6-Cl-THA relative to the separate monotherapies.

**Figure 2:**
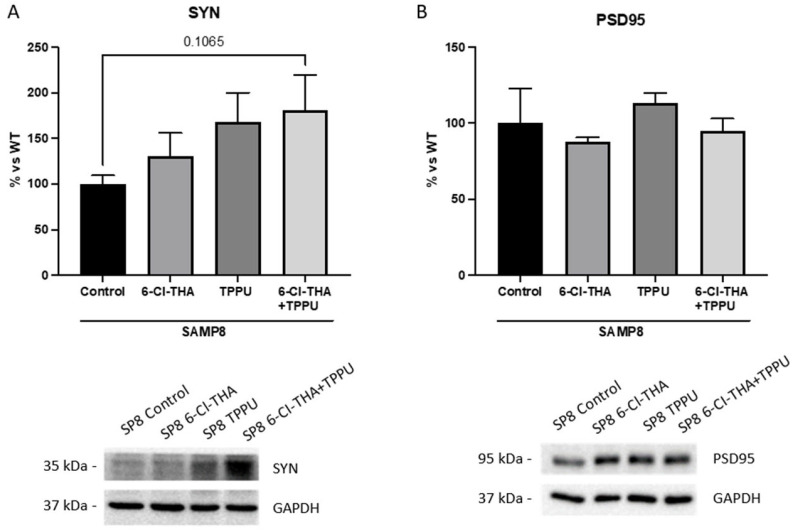
Neuronal synaptic markers in the SAMP8 hippocampus after treatments. Representative WB and quantification of (**A**) SYN and (**B**) PSD95. Results are expressed as a mean ± SEM. SAMP8 control, *n* = 3; SAMP8 treated with the TPPU (1 mg/kg/day), *n*= 3; SAMP8 treated with 6-Cl-THA (1 mg/kg/day), *n* = 3; SAMP8 subjected to co-treatment with TPPU (1 mg/kg/day) plus 6-Cl-THA (1 mg/kg/day), *n* = 3. Groups were compared by a one-way ANOVA test and post-hoc Tukey’s test. *n* = 4–6. Abbreviations: 6-Cl-THA = 6-chlorotacrine; SYN = synaptophysin; PSD95 = postsynaptic density protein; SP8 = SAMP8.

As aforementioned, synaptic plasticity and neuroinflammation are associated with cognitive decline. In addition, the dual inhibition of sEH and AChE was conceived to rescue neuroinflammation. Thus, we next evaluated synaptic and inflammatory markers to determine whether chronic low-dose sEHi/AChEi combination therapy could revert them in SAMP8. Firstly, we assessed synaptophysin (SYN) protein levels by using WB. Notably, we found increased SYN protein levels in the co-treatment group compared to the control SAMP8 mice, whereas the levels of SYN in the monotherapy groups were similar to those found in the co-treatment group (Figure 2A). Regarding the effects on postsynaptic density protein 95 (PSD95) protein levels, only a slight tendency towards increased PSD95 levels was observed in all SAMP8 treatment groups in comparison with the SAMP8 control group, even though it was not statistically significant (Figure 2B).

**Figure 3:**
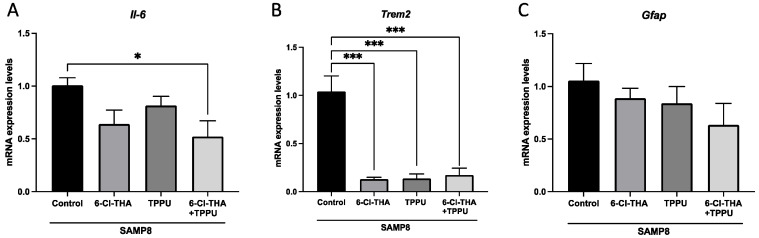
Gene expression of (**A**) *Il-6*, (**B**) *Trem2* and (**C**) *Gfap*. Results are expressed as a mean ± SEM. Groups were compared by the one-way ANOVA test and post-hoc Tukey’s test. SAMP8 control, *n* = 4; SAMP8 treated with the TPPU (1 mg/kg/day), *n* = 4; SAMP8 treated with 6-Cl-THA (1 mg/kg/day), *n* = 4; SAMP8 subjected to co-treatment with TPPU (1 mg/kg/day) plus 6-Cl-THA (1 mg/kg/day), *n* = 4. * *p* < 0.05; *** *p* < 0.001. Abbreviations: 6-Cl-THA = 6-chlorotacrine; *Il-6* = interleukin-6; *Trem2* = triggering receptor expressed on myeloid cell 2; *Gfap* = glial fibrillary acidic protein.

Moreover, we evaluated neuroinflammatory makers by qPCR. Strikingly, gene expression of interleukin-6 (*Il-6*), a pro-inflammatory cytokine, diminished in SAMP8 treated groups, with the decrease reaching only significance in the group of mice co-treated with TPPU plus 6-Cl-THA (Figure 3A). Accordingly, gene expression for microglial and astroglial markers, such as triggering receptor expressed on myeloid cell 2 *(Trem2)* and glial fibrillary acidic protein *(Gfap)*, was reduced in all treatment groups in comparison with the SAMP8 control group, albeit without significant differences among them (Figure 3B,C).

Therefore, in the LOAD mouse model, cognition and neuroinflammation were ameliorated after the chronic oral low-dose combination therapy, especially regarding the enhancement of long-term and spatial memory and the reduction of gene expression of the pro-inflammatory cytokine *Il-6*, for which combination therapy was superior to each separate monotherapy. As additional beneficial effects, sEHi/AChEi combination therapy, also led to a drastic reduction of gene expression of the *Trem2* and to a tendency towards diminished levels of *Gfap* and increased levels of the synaptic protein SYN, even though these effects were similar to those reached with monotherapies.

### 2.2. TPPU and Riv Chronic Low-Dose Co-Treatment Prevented 5XFAD Cognitive Decline, Improving Synaptic Markers and Ameliorating Neuroinflammation

**Figure 4:**
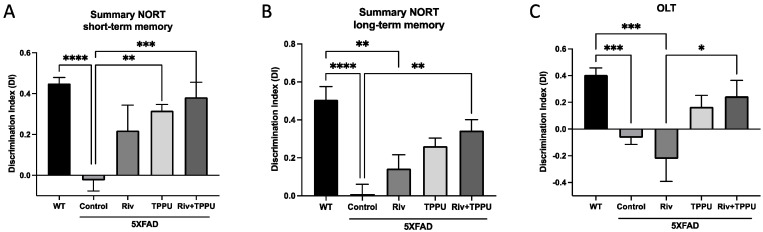
Effects of drug combination and monotherapies on cognitive performance in 5XFAD mice. (**A**) Short-term memory test at 2 h. (**B**) Long-term memory test at 24 h. (**C**) OLT. Results are expressed as a mean ± SEM. Groups were compared by the one-way ANOVA test and post-hoc Tukey’s test. WT, *n* = 12; 5XFAD control, *n* = 10; 5XFAD treated with TPPU (1 mg/kg/day), *n* = 8; 5XFAD treated with Riv (1 mg/kg/day), *n* = 8; 5XFAD subjected to co-treatment with TPPU (1 mg/kg/day) plus Riv (1 mg/kg/day), *n* = 8. * *p* < 0.05; ** *p* < 0.01; *** *p* < 0.001; **** *p* < 0.0001. Abbreviations: Riv = rivastigmine; NORT = novel object recognition test; OLT = object location test.

To further investigate the beneficial effects of the low dose oral administration of sEHi/AChEi combinations, we assessed in 5XFAD mice as a model of EOFAD, the dual treatment with the reference sEHi TPPU and the pseudoirreversible AChEi Riv. In general, all the treatment groups showed improved memory compared with the non-treated 5XFAD control mice, but only in the case of the sEHi/AChEi combination therapy the enhancement of the working and spatial memory reach significance in all the tests (Figure 4A–C), which indicated similar neuroprotective effects as those observed in SAMP8 mice. In SAMP8 mice, low-dose 6-Cl-THA could not revert either working or spatial memory (Figure 1A-C). Likewise, the same low dose of Riv could not ameliorate cognition, especially spatial memory, in 5XFAD, whereas TPPU prevented memory loss, reaching significance only in short-term memory (Figure 4A). Therefore, at the low doses tested, co-administration of Riv and TPPU potentiated the beneficial effects found for each compound in mono-therapy.

**Figure 5:**
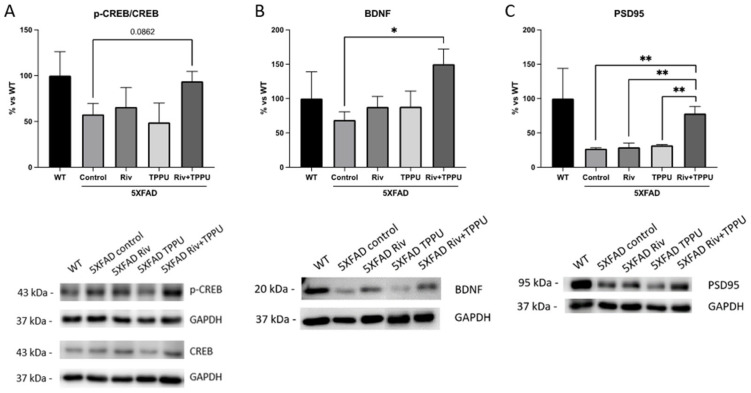
Effects of drug combination and monotherapies on neuronal plasticity in 5XFAD mice. Representative WB and quantification of (**A**) p-CREB/CREB ratio, (**B**) BDNF and (**C**) PSD95. Results are expressed as a mean ± SEM. WT, *n* = 3; 5XFAD control, *n* = 3; 5XFAD treated with TPPU (1 mg/kg/day), *n* = 3; 5XFAD treated with Riv (1 mg/kg/day), *n* = 3; 5XFAD subjected to co-treatment with TPPU (1 mg/kg/day) plus Riv (1 mg/kg/day), *n* = 4. Groups were compared by the one-way ANOVA test and post-hoc Tukey test. * *p* < 0.05; ** *p* < 0.01. Abbreviations: Riv = rivastigmine; CREB = cAMP response element-binding protein; BDNF = brain-derived neurotrophic factor; PSD95 = postsynaptic density protein.

As before, neuronal plasticity markers were examined to shed light on the potential mechanisms underlying the positive effects on cognitive performance. The cAMP response element-binding protein (CREB) is one of the major regulators of neurotrophins responses since phosphorylated CREB binds to a specific sequence in the promoter of brain-derived neurotrophic factor (BDNF) [29], and it is implicated in the learning and memory process [30]. On the one hand, as we expected, the ratio (p-CREB/CREB) was reduced in 5XFAD control in comparison with WT mice group (Figure 5A). However, neither TPPU nor Riv were able to increase CREB protein levels, while sEHi/AChEi co-treatment led to a tendency towards increased p-CREB/CREB ratio (Figure 5A). On the other hand, when BDNF protein levels were measured, only co-treatment was able to increase in a significant way the protein levels, indicating the CREB pathway activation (Figure 5B). Likewise, neither Riv nor TPPU were able to increase PSD95 protein levels, but co-treatment did in a significant way (Figure 5C). These results highlight the potential of sEHi/AChEi combination therapies as a novel strategy to improve neuronal plasticity and cognition in 5XFAD mice.

**Figure 6:**
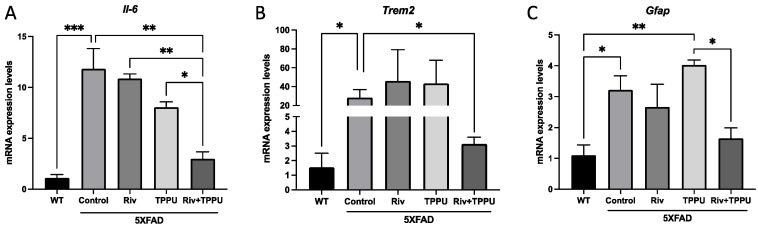
Gene expression of (**A**) *Il-6*, (**B**) *Trem2* and (**C**) and *Gfap*. Results are expressed as a mean ± SEM. WT, *n* = 4; 5XFAD control, *n* = 3; 5XFAD treated with TPPU (1 mg/kg/day), *n* = 3; 5XFAD treated with Riv (1 mg/kg/day), *n* = 3; 5XFAD subjected to co-treatment with TPPU (1 mg/kg/day) plus Riv (1 mg/kg/day), *n* = 4. Groups were compared by the one-way ANOVA test and post-hoc Tukey test. * *p* < 0.05; ** *p* < 0.01; *** *p* < 0.001. Abbreviations: *Riv* = rivastigmine; *Il-6* = interleukin-6; *Trem2* = triggering receptor expressed on myeloid cell *2; Gfap =* glial fibrillary acidic protein.

We next studied the neuroinflammatory markers by qPCR. Expectedly, gene expression for *Il-6*, *Trem2* and *Gfap* increased in 5XFAD control mice relative to WT mice, showing microgliosis and astrogliosis (Figure 6A-C). Very interestingly, low-dose co-treatment with TPPU and Riv led to a significant decrease in gene expression of the pro-inflammatory cytokine *Il-6* as well as *Trem2* and *Gfap* compared to the 5XFAD control group, whereas none of the monotherapies was able to induce any change (Figure 6A–C). Furthermore, in case of *Il-6* gene expression, we found a significant reduction among the 5XFAD low-dose co-treatment and the other treated groups (Figure 6A). These results suggest an apparent synergistic effect at the molecular level targeting both sEH and AChE.

## 3. Discussion

AD drug discovery is facing one of the highest attrition rates [7]. Currently, only four drugs have reached global regulatory approval, i.e., the AChE inhibitors donepezil, Riv and galantamine and the glutamate NMDA receptor antagonist memantine. These drugs are regarded as symptomatic and, hence, are used to treat the cognitive and functional deficits of AD patients, offering a limited temporary effect [31]. Since the approval of memantine in 2003, huge efforts and investments have been done for the development of new drugs that selectively and potently modulate one particular biological target with a key role in AD pathogenesis, with the hope that they could alter disease progression. Most of these strategies have been directed to the β-amyloid peptide, but other targets and pathological events, such as Tau protein, oxidative stress and neuroinflammation, among others, have also been pursued. Thus far, more than 2000 clinical trials, mostly involving potentially disease-modifying single-target drug candidates, have been performed [32]. Unfortunately, these titanic endeavors have met with minimal success. In 2019, the marine algae-derived sodium oligomannate, which seems to act via the gut–brain axis, was conditionally approved in China, but it has not yet been approved in USA and Europe, where it is undergoing Phase III clinical trials [33]. In 2021, the β-amyloid-directed monoclonal antibody aducanumab was approved by the Food and Drug Administration (FDA), but not by the European Medicines Agency (EMA) due to lack of efficacy and safety issues [34].

The distressing panorama of AD drug discovery calls for new design paradigms based on unexplored biological targets and/or new design strategies. In this context, AD is being increasingly conceived as the result of a robust multifactorial pathogenic network, in which not just one but several biological targets play a key role. This complex network would remain resistant to the modulation of any single biological target. Conversely, simultaneous modulation of several targets (multitarget approach) has emerged as a very promising option to efficiently cope with the AD pathological network [35]. Very intensive research has been done in the last decade for the development of different classes of multitarget agents, i.e., single molecules designed to hit several biological targets [36]. However, much less attention is being given to the development of combination therapies, i.e., combinations of two or more drugs each one hitting a different biological target. The latter approach has been successfully applied to other multifactorial diseases, such as cancer and HIV [21]. Not only have new drug combinations may have a therapeutic interest by themselves, but they can also be used to experimentally validate a multitarget approach, which can support a dual mechanism of action of new classes of multitarget compounds [37].

In this context, we and others have validated sEH as a new biological target for AD treatment [12,38,39]. This enzyme is upregulated in the brains of AD patients and its inhibition results in cognition-enhancing effects and amelioration of neuroinflammation, oxidative stress and neuronal apoptosis, among other critical neuropathological processes, in different mouse models of AD [12,17,19,40]. Very interestingly, sEH has not been pursued in any anti-AD multitarget strategy until very recently, when we developed a novel class of dual inhibitors of sEH and AChE [19]. The rationale behind such a combination of sub-optimal neuroprotective doses for each compound lies in describing the possible potentiation of the anti-neuroinflammatory effects of EETs, whose levels should increase as a consequence of both sEH inhibition and activation of muscarinic M1 receptors by the increased levels of ACh resulting from AChE inhibition [20], besides the pro-cognitive effects arising from the cholinergic effect. Remarkably, the neuroprotective range doses of TPPU (1 to 5 mg/kg) [12], 6-Cl-THA (0.9 to 5 mg/kg) [41] and Riv (0.6 to 2.5 mg/kg) [42] are well established. Furthermore, chronic oral administration of a low dose (2 mg/kg/day) of this lead compound rescued memory, synaptic plasticity and neuroinflammation in SAMP8 mice [19]. Interestingly, the dual sEH/AChE inhibitor lead was designed by molecular hybridization of the pharmacophores of the reference sEHi TPPU [12] and the potent reversible AChEi 6-Cl-THA [11].

Given (i) the novelty and relevance of sEH and the combination of sEH/AChE inhibition for the development of new therapies for AD; (ii) the scarcity of combination therapies for AD; (iii) the therapeutic potential of combination therapies to tackle multifactorial disease; and (iv) the usefulness of the experimental confirmation of the beneficial effects of drug combinations to fuel the development of novel classes of multitarget agents hitting the same combination of targets, in this work, we have explored the effects on memory and molecular markers of synaptic plasticity and neuroinflammation of the chronic (four-week) low-dose (1 + 1 mg/kg/day) oral administration of a combination of sEHi/AChEi. To this end, we have used TPPU as a standard sEHi and either a reversible (6-Cl-THA) or a pseudoirreversible (Riv) AChEi and two different mouse models of AD, a model of late-onset AD (SAMP8) and a model of early-onset familial AD (5XFAD). In each case, we have compared the effects exerted by the co-treatment with those produced by the corresponding separate monotherapies at a dose of 1 mg/kg/day.

The SAMP8 is an accelerated aging model established through phenotypic selection from AKR/J mice. SAMP8 exhibits cognitive and behavioral abnormalities from young ages [43,44]. Likewise, this murine model shows histopathological features of LOAD, such as abnormal APP and Aβ processing and Tau pathology, and inflammatory and oxidative stress markers [44]. In this first experiment, we wanted to know the neuroprotective effects in SAMP8 without comparing with its healthy control strain the senescence-resistant inbred strain 1 (SAMR1). In this model, the studied low dose of the monotherapies 6-Cl-THA and TPPU did not deliver any significant beneficial effect, on cognition or synaptic markers. However, monotherapies were uniquely able to reduce microgliosis, as evidenced by the significantly reduced levels of the *Trem2* marker. Interestingly, equal low doses of co-treatment with 6-Cl-THA (1 mg/kg/day) plus TPPU (1 mg/kg/day) significantly improved cognitive performance in all tests, indicating a synergistic beneficial effect. Gratifyingly, a significant diminution of neuroinflammation and astrogliosis was also found in the group of mice treated with the combination of drugs. However, the combination treatment only led to a subtle beneficial effect on synaptic markers, such as SYN and PSD95, suggesting that an increase in the selected dose or a different timing and duration of the pharmacological intervention might have led to more robust effects on synaptic plasticity.

The 5XFAD mouse is a well-established model of EOFAD, carrying human APP and PSEN1 transgenes with a total of five AD-linked mutations: the Swedish (K670N/M671L), Florida (I716V) and London (V717I) mutations in APP, and the M146L and L286V mutations in PSEN1 [45]. Then, this model also enables the assessment of a wide range of responses to drugs against AD, including cognitive readouts and synaptic and neuroinflammatory markers. Again, neither the AChEi (Riv) nor the sEHi (TPPU) in monotherapy at a dose of 1 mg/kg/day were able to significantly ameliorate cognition in any memory paradigm, with the sole exception of TPPU in the NORT short-term memory assay. In contrast, the combination sEHi/AChEi (1 + 1 mg/kg/day) significantly improved cognition in all the tests. The used low doses of Riv and TPPU are under the therapeutic range, which accounts for the lack of effects of monotherapies and, at the same time, confirms that the co-administration of both drugs leads to a potentiation rather than just an additive effect. Regarding the molecular markers, the sEHi/AChEi co-treatment led to a positive tendency towards an increased p-CREB/CREB ratio and significantly increased protein levels of the synaptic plasticity markers BDNF. As mentioned, BDNF is a neurotrophic factor involved in neuronal plasticity and is considered a master key in the synaptic function. Additionally, increased protein levels of PSD95, a marker of synaptic formations, confirmed again a synergistic effect produced by the combination of drugs, clearly superior to that of the monotherapies, which separately did not lead to beneficial effects at the studied low dose. The beneficial effects of the combination of drugs may partly explain the prevention of cognitive decline and the potentiation effect of this beneficial effect in 5XFAD. Finally, the co-treatment, but not the monotherapies, led to a significant reduction of the neuroinflammation markers, *Il-6*, *Trem2* and *Gfap* compared to the non-treated 5XFAD control mice, thereby confirming the pursued synergistic effect on neuroinflammation.

## 4. Materials and Methods

### 4.1. Animals and Treatments

**Figure 7:**
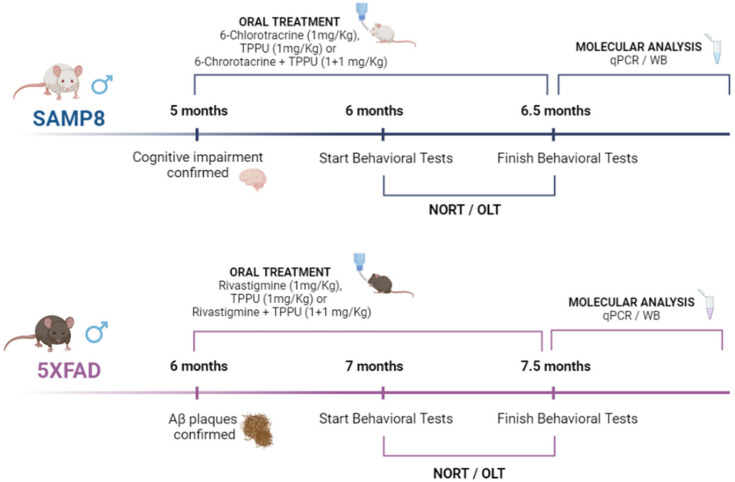
Experimental design scheme. SAMP8 control; SAMP8 treated with the TPPU (1 mg/kg/day); SAMP8 treated with 6-Cl-THA (1 mg/kg/day), SAMP8 subjected to co-treatment with TPPU (1 mg/kg/day) plus 6-Cl-THA (1 mg/kg/day). 5XFAD control; 5XFAD treated with TPPU (1 mg/kg/day), 5XFAD treated with Riv (1 mg/kg/day); 5XFAD subjected to co-treatment with TPPU (1 mg/kg/day) plus Riv (1 mg/kg/day). Abbreviations: 6-Cl-THA: 6-chlorotacrine; Riv = rivastigmine.

In the study, 5-month-old male SAMP8 mice (*n* = 32) and 6-month-old male 5XFAD (*n* = 34) were used to perform behavioral and molecular analyses. Moreover, 6-month-old male (*n* = 12) wild type (WT) mice were also added to 5XFAD experiments. SAMP8 were randomly divided into control (*n* = 8), SAMP8 treated with the sEHi TPPU (*n* = 8, 1 mg/kg/day), SAMP8 treated with the reversible AChEi 6-Cl-THA (*n* = 8, 1 mg/kg/day) and SAMP8 subjected to co-treatment with TPPU plus 6-Cl-THA (*n* = 8, 1 + 1 mg/kg/day). 5XFAD were randomly divided into 5XFAD control (*n* = 10), 5XFAD treated with the sEHi TPPU (*n* = 8, 1 mg mg/kg/day), 5XFAD treated with the pseudoirreversible AChEi Riv (*n* = 8, 1 mg/kg/day) and 5XFAD subjected to co-treatment with TPPU plus Riv (*n* = 8, 1 + 1 mg/kg/day). The animals had free access to food and water and were kept under standard temperature conditions (22 ± 2 °C) and 12-h/12-h light/dark cycles (300 lux/0 lux). Compounds were dissolved in 1.8% (2-hydroxypropyl)-β-cyclodextrin (Sigma-Aldrich, St. Louis, MO, USA) and administered through drinking water for 4 weeks (Figure 7). Control groups (SAMP8, 5XFAD and WT) received water plus 1.8% (2-hydroxypropyl)-β-cyclodextrin during the treatment period. After 4 weeks of treatment, behavioral and cognitive tests were performed to study the effects of treatment on working and spatial memory. Drug administration was continued until euthanasia by cervical dislocation, three days after the cognitive tests were completed. The brains were immediately removed from the skulls and the hippocampi were dissected, frozen and maintained at −80 °C. Weight and water consumption were controlled each week, and compound concentration was adjusted accordingly to reach the optimal dose. Mice were treated according to European Community Council Directive 86/609/EEC and the studies were approved by the Institutional Animal Care and Use Committee of the University of Barcelona (670/14/8102) and by the Generalitat de Catalunya, Spain (10291). All studies and procedures for the behavioral tests, brain dissection and extractions followed the ARRIVE. Every effort was made to minimize animal suffering and reduce the number of animals.

### 4.2. Behavioral Tests

The Novel Object Recognition Test (NORT) protocol was performed as follows: mice were placed in a 90° two-arm (25 × 20 × 5 cm) black maze, with removable walls for easy cleaning and light intensity in mid-field was 30 lux. Before the memory trials, mice were habituated to the apparatus for 10 min for 3 days. On day 4, the animals were subjected to a 10 min acquisition trial, in which they were allowed to freely explore two identical objects located at the end of each arm (First trial—Familiarization). After 2 h (short-term memory determination) and 24 h (long-term memory determination) from the first trial, mice were subjected to a 10 min retention trial, in which one of the two old objects had been replaced by a novel one. The behavior was recorded, and the time that the mice spent exploring the new object (TN) and the old one (TO) were measured manually. Exploration was defined as sniffing or touching objects with the nose and/or forepaws. The discrimination index (DI) was calculated as (TN − TO)/(TN + TO). To avoid object preference biases, objects were alternated. In total, 70% EtOH was used to clean the arms and objects after each trial for the elimination of olfactory cues.

The Object Novel Location Test (OLT) was performed in a cage (50 × 50 × 25 cm), in which three walls were white and one was black, and lasted 3 days. On day 1, mice were familiarized with the arena for 10 min. On day 2, two identical objects (A + A) were in front of the black wall, and the mice were freely allowed to explore both objects for 10 min (Trial 1—training phase). On day 3, after a retention period of 24 h, mice were returned to the testing arena for another 10 min (Trial 2—testing phase), with one object moved to a different position (opposite direction toward the black wall) and were allowed to explore. The trials were recorded, and the object exploration time was measured manually. In addition, the time sniffing the object in the old position (TO) and the time exploring the object in the new position (TN) were evaluated.

The DI defined as (TN − TO)/(TN + TO) was determined as an indicator of cognitive performance. For the elimination of olfactory cues, 70% EtOH was used to clean the testing arena after each trial.

### 4.3. Immunodetection Experiments by Western Blotting

The hippocampal tissue from each animal was homogenized in lysis buffer (Tris HCl pH 7.4 mM, NaCl 150 mM, EDTA 5 mM and 1X-Triton X-100) containing phosphatase and protease inhibitors (Cocktail II, Sigma-Aldrich, St. Louis, MO, USA) to obtain total protein homogenates. The Bradford technique was used to determine total protein concentration. Aliquots of 15 μg of total hippocampal protein were used and separated by sodium dodecyl sulfate-polyacrylamide gel electrophoresis (SDS-PAGE) (8–20%) and transferred into polyvinylidene difluoride (PVDF) membranes (Merck-Millipore, Burlington, MA, USA) for 2 h. The membranes were blocked in 5% non-fat milk in TRIS-buffered saline (TBS) containing 0.1% Tween 20 (TBS-T, Sigma-Aldrich, St. Louis, MO, USA) for 1 h at room temperature, and then, overnight incubation at 4 °C with the primary antibodies listed in Table S1 (Supplementary Materials) was carried out. The membranes were washed with TBS-T 3 times for 5 min and incubated with secondary antibodies for 1 h at room temperature. Immunoreactive protein was viewed with a Chemiluminescence-based detection kit, following the manufacturer’s protocol (ECL Kit; Merck-Millipore, Billerica, MA, USA), and digital images were acquired using a ChemiDoc XRS+ System (BioRad Lab, Hercules, CA, USA). Semi-quantitative analyses were done using ImageLab Software (BioRad Lab, Hercules, CA, USA), and results were expressed in Arbitrary Units (AU), considering control protein levels as 100%. Protein loading was routinely monitored by immunodetection of glyceraldehyde-3-phosphate dehydrogenase (GADPH).

### 4.4. RNA Extraction and Gene Expression Determination by qPCR

Total RNA isolation from hippocampal tissue was carried out using TRIsure™ reagent following the manufacturer’s instructions (Bioline Reagents, London, UK). The yield, purity and quality of RNA were determined spectrophotometrically with a NanoDrop™ ND-1000 (Thermo Fisher Scientific, Waltham, MA, USA) apparatus and an Agilent 2100B Bioanalyzer (Agilent Technologies, Santa Clara, CA, USA). RNAs with 260/280 ratios and RIN higher than 7.5, respectively, were selected (Table S2, Supplementary Materials). Reverse transcription-polymerase chain reaction (RT-PCR) was performed as follows: 2 μg of messenger RNA (mRNA) was reverse-transcribed using the high-capacity cDNA reverse transcription Kit (Applied Biosystems, Foster City, CA, USA). Real-time quantitative PCR (qPCR) was used to quantify mRNA expression genes listed in Table S3 (Supplementary Materials). Real-time PCR was performed by using Step One Plus Detection System (Applied Biosystems, Waltham, MA, USA) employing SYBR^®^ Green PCR Master Mix (Applied Biosystems) (Table S4). Each reaction mixture contained 6.75 μL of complementary DNA (cDNA) (2 μg μL^−1^), 0.75 μL of each primer (100 nM) and 6.75 μL of SYBR^®^ Green PCR Master Mix (2X) (Applied Biosystems). Data were analyzed utilizing the comparative Cycle threshold (Ct) method (ΔΔCt), where the housekeeping gene level was used to normalize differences in sample loading and preparation. Normalization of expression levels was performed with β-actin for SYBR^®^ Green-based real-time PCR results. Each sample was analyzed in triplicate and the results represent the n-fold difference in the transcript levels among groups.

### 4.5. Statistical Analysis

Data analysis was conducted using GraphPad Prism ver. 9 statistical software. Data are expressed as the mean ± standard error of the mean (SEM) of at least eight samples per group for behavioral tests and four samples for molecular techniques. Data have been analyzed using the Shapiro–Wilk test for normality to ensure that parametric tests can be used. One-way analysis of variance (ANOVA) was followed by Tukey post-hoc analysis or two-tail Student’s t-test when necessary. Statistical significance was considered when *p*-values were <0.05. The statistical outliers were determined with Grubbs’ test and, when necessary, were removed from the analysis.

## 5. Conclusions

In conclusion, our results in SAMP8 and 5XFAD mice indicated that the combined low-dose therapy of a sEHi plus an AChEi led to a better readout in cognitive tests than the corresponding separate monotherapies. Besides, molecular markers that underlie this cognition improvement, such as synaptic plasticity and neuroinflammation, were also positively modulated after the combination treatment but not with monotherapies. Globally, these results highlight the sEHi/AChEi combination therapy as a novel promising strategy to cope with important disease mechanisms and cognitive symptoms of AD and validate the dual inhibition of both enzymes as a valuable mechanism of action of novel classes of multitarget compounds that are to come, thereby opening new avenues to AD therapy.

## Data Availability

Not applicable.

## Supplementary Materials

The following supporting information can be downloaded at: https://www.mdpi.com/article/10.3390/ph15080908/s1, Table S1: Antibody used in Western blot studies; Table S2: Quality parameters of all RNA extracted in this study. Table S3: Primers sequences of real-time qPCR (SYBR Green primers); Table S4. RT-qPCR cycling conditions.
