# Supplementary material for: A Combined Chronic Low-Dose Soluble Epoxide Hydrolase and Acetylcholinesterase Pharmacological Inhibition Promotes Memory Reinstatement in Alzheimer’s Disease Mice Models
Source: Pharmaceuticals (Basel). 2022 Jul 22;15(8):908. doi: 10.3390/ph15080908 (PMC9394299; doi:10.3390/ph15080908)
Supplement: Supplementary file 1 [file pharmaceuticals-15-00908-s001.zip › pharmaceuticals-1818791-supplementary.pdf]

Table S1. Antibodies used in Western blot studies.

Antibody	Host	Source/Catalog	WB dilution
**SYN**	Rabbit	Dako/CloneSY38	1:1000
**p-CREB (Ser133)**	Rabbit	Cell Signalling/#9198	1:1000
**CREB**	Rabbit	Cell Signaling/#4820	1:1000
**BDNF**	Rabbit	Bios/BS-4989R	1:1000
**PSD95**	Rabbit	Abcam/ab18258	1:1000
**GAPDH**	Mouse	Millipore/MAB374	1:5000
**Donkey-anti-goat HRP conjugated**		Santa Cruz/sc-2020	1:2000
**Goat-anti-rabbit HRP conjugated**		Biorad/170-6515	1:2000
**Goat-anti-mouse HRP conjugated**		Biorad/170-5047	1:2000

Table S2. Quality parameters of all RNA extracted in this study.

Animal	Concentration (ng/µL)	260/280	RIN
SAMP8 Ct 1	497.10	2.04	8.1
SAMP8 Ct 2	665.00	2.06	8.0
SAMP8 Ct 3	782.10	2.09	7.7
SAMP8 Ct 4	755.32	2.10	7.9
SAMP8 6-Cl-THA 1	412.30	2.05	8.0
SAMP8 6-Cl-THA 2	879.10	2.04	7.9
SAMP8 6-Cl-THA 3	557.30	2.00	7.6
SAMP8 6-Cl-THA 4	679.12	2.01	7.6
SAMP8 TPPU 1	801.20	2.03	7.7
SAMP8 TPPU 2	698.10	2.03	7.7
SAMP8 TPPU 3	573.40	2.05	8.1
SAMP8 TPPU 4	891.03	2.02	8.9
SAMP8 6-Cl-THA + TPPU 1	327.80	2.04	7.8
SAMP8 6-Cl-THA + TPPU 2	521.70	2.02	8.2
SAMP8 6-Cl-THA + TPPU 3	887.00	2.01	8.5
SAMP8 6-Cl-THA + TPPU 4	903.45	2.10	8.0
WT 1	418.00	2.08	8.1
WT 2	588.10	2.13	7.5
WT 3	574.10	1.99	7.5
WT 4	572.60	1.99	7.6
5XFAD Ct 1	560.20	2.05	8.2
5XFAD Ct 2	685.60	2.05	7.6
5XFAD Ct 3	872.70	2.06	8.4
5XFAD TPPU 1	449.60	2.05	8.4
5XFAD TPPU 2	399.40	2.00	8.1
5XFAD TPPU 3	994.00	2.11	7.6
5XFAD RIV 1	1244.00	2.10	7.7
5XFAD RIV 2	787.60	1.99	7.9
5XFAD RIV 3	632.10	2.00	8.4
5XFAD RIV + TPPU 1	463.10	2.00	7.9
5XFAD RIV + TPPU 2	1552.40	2.07	8.5
5XFAD RIV + TPPU 3	300.80	2.00	8.5
5XFAD RIV + TPPU 4	476.60	2.10	8.2

Table S3. Primer sequences of real-time qPCR (SYBR Green primers).

Target	Forward primer (5′-3′)	Reverse primer (5′-3′)	Product length
Il-6	ATCCAGTTGCCTTCTTGGGACTGA	TAAGCCTCCGACTTGTGAAGTGGT	134
Trem2	CCTGAAGAAGCGGAATGGG	CTTGATTCCTGGAGGTGCT	269
Gfap	CCTTCTGACACGGATTTGGT	ACATCGAGATCGCCACCTAC	247
β-actin	CAACGAGCGGTTCCGAT	GCCACAGGTTCCATACCCA	66

Table S4. RT-qPCR cycling conditions.

Step	Temperature, °C	Time	Number of cycles
Initial denaturation	95	10 min	1
Denaturation	95	15 s	40
Annealing	60	30 s	
Extension	72	30 s	
